# Supplementary material for: DNA Damage Response−Related Proteins Are Prognostic for Outcome in Both Adult and Pediatric Acute Myelogenous Leukemia Patients: Samples from Adults and from Children Enrolled in a Children’s Oncology Group Study
Source: Int J Mol Sci. 2023 Mar 20;24(6):5898. doi: 10.3390/ijms24065898 (PMC10058043; doi:10.3390/ijms24065898)
Supplement: Supplementary file 1 [file ijms-24-05898-s001.zip › Supplemental Tables/Supplemental Table S3.pdf]

**Supplemental Table S3.** DDR-related proteins are prognostic for overall survival in patients receiving venetoclax + HMA therapy (VH).

| Patients receiving venetoclax plus hypomethylating agent (HMA) therapy (VH) | | | | | |
|---|---|---|---|---|---|
| Protein Name | Overall survival stratifications | | | | |
| | Median Split | Terciles | Quartiles | Quintiles | Sextiles |
| SSBP2 | 0.026 | 0.18 | 0.16 | 0.074 | 0.19 |
| MSH6 | 0.62 | 0.2 | 0.022 | 0.45 | 0.2 |
| TP53BP1 | 0.038 | 0.38 | 0.18 | 0.4 | 0.29 |
| RAD51 | 0.022 | 0.24 | 0.077 | 0.21 | 0.2 |
| CHEK2 | 0.0013 | 0.058 | 0.011 | 0.014 | 0.026 |
| SIRT6 | 0.021 | 0.23 | 0.15 | 0.076 | 0.27 |
| PDCD1 | 0.033 | 0.082 | 0.2 | 0.17 | 0.076 |
| TP53 | 0.81 | 0.2 | 0.79 | 0.78 | 0.029 |
| CDK1 | 0.43 | 0.13 | 0.038 | 0.18 | 0.38 |
| CDK1_2_3.pT14 | 0.0098 | 0.0078 | 0.049 | 0.02 | 0.055 |
| CDK9 | 0.041 | 0.0052 | 0.14 | 0.071 | 0.038 |
| CDKN1B | 0.0001 | 0.0001 | 0.0001 | 0.0001 | 0.00016 |
| CDKN1B.pS10 | 0.0005 | 0.00016 | 0.00023 | 0.00044 | 0.0001 |
| CDKN1B.pT198 | 0.0038 | 0.03 | 0.028 | 0.12 | 0.085 |
| RPA32.pS4_8 | 0.026 | 0.092 | 0.12 | 0.14 | 0.25 |
