# Supplementary material for: DNA Damage Response−Related Proteins Are Prognostic for Outcome in Both Adult and Pediatric Acute Myelogenous Leukemia Patients: Samples from Adults and from Children Enrolled in a Children’s Oncology Group Study
Source: Int J Mol Sci. 2023 Mar 20;24(6):5898. doi: 10.3390/ijms24065898 (PMC10058043; doi:10.3390/ijms24065898)
Supplement: Supplementary file 1 [file ijms-24-05898-s001.zip › Supplemental Tables/Supplemental Table S2.pdf]

Supplemental Table S2. DDR-related proteins are prognostic for overall survival in patients receiving conventional chemotherapy (CC).

Patients receiving Conventional Chemotherapy (CC)					
Protein Name	Overall survival stratifications				
	Median Split	Terciles	Quartiles	Quintiles	Sextiles
XPF	0.017	0.00098	0.00069	0.036	0.0064
SSBP2	0.017	0.036	0.058	0.29	0.089
MSH6	0.016	0.013	0.028	0.0034	0.12
TP53BP1	0.0001	0.0029	0.00022	0.005	0.0022
RAD50	0.01	0.19	0.028	0.21	0.13
XRCC1	0.58	0.12	0.15	0.037	0.25
BABAM1.pS29	0.00037	0.00014	0.00083	0.00049	0.00057
CDKN1B.pT198	0.14	0.0087	0.037	0.068	0.062
AURKA	0.34	0.49	0.047	0.02	0.003
RPA32	0.0063	0.0052	0.022	0.0093	0.0095
RPA32.pS4_8	0.35	0.004	0.065	0.24	0.022
