# Supplementary material for: DNA Damage Response−Related Proteins Are Prognostic for Outcome in Both Adult and Pediatric Acute Myelogenous Leukemia Patients: Samples from Adults and from Children Enrolled in a Children’s Oncology Group Study
Source: Int J Mol Sci. 2023 Mar 20;24(6):5898. doi: 10.3390/ijms24065898 (PMC10058043; doi:10.3390/ijms24065898)
Supplement: Supplementary file 1 [file ijms-24-05898-s001.zip › Supplemental Tables/Supplemental Table S1.pdf]

Supplemental Table S1. Multivariate analysis for ExpDDR clusters.

	Univariate OS (N=810)			Multivariate OS (N=810)			Univariate CRD (N=398)			Multivariate CRD (N=398)		
**Variable**	**HR**[1]	**95% CI**[1]	**p-value**	**HR**[1]	**95% CI**[1]	**p-value**	**HR**[1]	**95% CI**[1]	**p-value**	**HR**[1]	**95% CI**[1]	**p-value**
**Cluster**												
C1	1.00	—		1.00	—		1.00	—		1.00	—	
C2	1.08	0.80, 1.44	0.62	0.51	0.35, 0.75	**<0.001**	1.23	0.77, 1.95	0.38	0.84	0.46, 1.55	0.58
C3	1.19	0.90, 1.58	0.22	0.54	0.38, 0.77	**<0.001**	1.09	0.68, 1.75	0.72	0.72	0.40, 1.31	0.28
C4	1.18	0.89, 1.56	0.24	0.59	0.42, 0.84	**0.003**	0.90	0.56, 1.45	0.66	0.68	0.36, 1.26	0.22
C5	1.31	0.98, 1.76	0.070	0.67	0.47, 0.97	**0.035**	1.22	0.75, 1.98	0.43	0.87	0.44, 1.73	0.69
C6	1.74	1.27, 2.37	**<0.001**	0.60	0.40, 0.89	**0.012**	1.44	0.84, 2.48	0.18	0.72	0.35, 1.49	0.38
**Age (years)**	1.03	1.03, 1.04	**<0.001**	1.03	1.02, 1.04	**<0.001**	1.04	1.03, 1.05	**<0.001**	1.03	1.01, 1.04	**<0.001**
**White (race)**	1.30	1.06, 1.60	**0.013**				1.19	0.84, 1.68	0.33			
**Asian (race)**	0.60	0.37, 0.97	**0.038**				1.08	0.55, 2.12	0.81			
**Hispanic (race)**	0.68	0.49, 0.95	**0.023**				0.71	0.42, 1.21	0.21			
**2nd AML**	2.38	2.01, 2.81	**<0.001**	1.45	1.16, 1.82	**0.001**	1.93	1.44, 2.58	**<0.001**	1.38	0.92, 2.05	0.12
**Unfav. Cyto. Risk**	2.24	1.89, 2.66	**<0.001**				1.71	1.27, 2.31	**<0.001**			
**Complex Kar.**	2.44	2.05, 2.91	**<0.001**	1.78	1.35, 2.35	**<0.001**	1.95	1.42, 2.68	**<0.001**	1.80	1.08, 3.01	**0.024**
**-5/5q-**	2.54	2.07, 3.12	**<0.001**				2.35	1.60, 3.45	**<0.001**			
**-7/7q-**	2.24	1.84, 2.74	**<0.001**				2.02	1.36, 3.01	**<0.001**			
**t(8;21)**	0.38	0.20, 0.72	**0.003**				0.68	0.34, 1.39	0.29			
**Inv 16**	0.12	0.04, 0.32	**<0.001**				0.25	0.09, 0.67	**0.006**			
**Del 12**	2.34	1.57, 3.48	**<0.001**				2.33	0.96, 5.68	0.062			
**Trisomy 6**	2.27	1.28, 4.02	**0.005**				1.08	0.27, 4.37	0.91			
**t(11q23)**	0.97	0.55, 1.71	0.91				2.23	1.04, 4.75	**0.038**			
**CEBPA Mut.**	0.54	0.39, 0.75	**<0.001**	0.75	0.53, 1.07	0.11	0.84	0.53, 1.33	0.46			
**FLT3 Mut.**	0.67	0.53, 0.85	**<0.001**				0.71	0.49, 1.02	0.067			
**DNMT3 Mut.**	0.77	0.62, 0.96	**0.022**				0.96	0.68, 1.36	0.82			
**KIT Mut.**	0.51	0.29, 0.91	**0.023**	0.68	0.36, 1.29	0.23	0.69	0.31, 1.56	0.37			
**RUNX1 Mut.**	1.24	0.96, 1.60	0.11				1.89	1.19, 3.00	**0.007**	1.76	1.05, 2.95	**0.032**
**IDH2 Mut.**	0.64	0.49, 0.84	**0.001**	0.79	0.57, 1.09	0.15	0.65	0.41, 1.01	0.057			
**JAK2 Mut.**	1.97	1.37, 2.84	**<0.001**				1.61	0.75, 3.42	0.22			
**NPM1 Mut.**	0.58	0.45, 0.75	**<0.001**	0.72	0.52, 0.98	**0.039**	0.47	0.31, 0.70	**<0.001**	0.51	0.29, 0.90	**0.021**
**SRSF2 Mut.**	0.51	0.31, 0.84	**0.008**				0.60	0.27, 1.35	0.22			
**TP53 Mut.**	2.89	2.37, 3.53	**<0.001**	1.44	1.07, 1.95	**0.017**	2.62	1.83, 3.77	**<0.001**	1.30	0.73, 2.32	0.37

[1] HR = Hazard Ratio, CI = Confidence Interval
