# Supplementary material for: DNA Damage Response−Related Proteins Are Prognostic for Outcome in Both Adult and Pediatric Acute Myelogenous Leukemia Patients: Samples from Adults and from Children Enrolled in a Children’s Oncology Group Study
Source: Int J Mol Sci. 2023 Mar 20;24(6):5898. doi: 10.3390/ijms24065898 (PMC10058043; doi:10.3390/ijms24065898)
Supplement: Supplementary file 1 [file ijms-24-05898-s001.zip › Supplemental Tables/Supplemental Table S5.pdf]

Supplemental Table S5. Multivariate analysis for VH clusters.

	Univariate OS (N=79)			Multivariate OS (N=79)			Univariate CRD (N=51)			Multivariate CRD (N=51)		
**Variable**	**HR[1]**	**95% CI[1]**	**p-value**	**HR[1]**	**95% CI[1]**	**p-value**	**HR[1]**	**95% CI[1]**	**p-value**	**HR[1]**	**95% CI[1]**	**p-value**
**Cluster**												
C1	1.00	—		1.00	—		1.00	—		1.00	—	
C2	3.21	1.79, 5.75	**<0.001**	3.87	1.65, 9.07	**0.002**	2.26	0.93, 5.49	0.073	1.59	0.60, 4.24	0.35
C3	3.99	1.97, 8.07	**<0.001**	4.79	1.60, 14.4	**0.005**	4.28	1.44, 12.7	**0.009**	4.51	1.27, 16.0	**0.020**
**Age (years)**	1.01	0.98, 1.04	0.68	0.97	0.92, 1.04	0.42	1.09	1.02, 1.16	**0.012**	1.10	1.02, 1.19	**0.016**
**2nd AML**	2.03	1.09, 3.76	**0.025**	1.89	0.82, 4.37	0.14	2.03	0.81, 5.11	0.13	1.33	0.47, 3.71	0.59
**Unfav. Cyto. Risk**	2.27	1.33, 3.88	**0.003**				2.36	1.07, 5.19	**0.033**			
**Complex Kar.**	3.10	1.78, 5.39	**<0.001**	0.50	0.12, 2.10	0.34	3.75	1.65, 8.52	**0.002**	2.45	0.97, 6.18	0.057
**FLT3 Mut.**	1.98	0.85, 4.59	0.11				6.08	1.43, 26.0	**0.015**			
**IDH Mut.**	0.34	0.16, 0.71	**0.004**	0.21	0.08, 0.61	**0.004**	0.43	0.17, 1.12	0.084			
**RAS Mut.**	1.44	0.68, 3.05	0.34				3.11	1.13, 8.55	**0.027**			
**PTPN11 Mut.**	7.47	2.13, 26.2	**0.002**	13.4	2.23, 80.6	**0.005**						
**SRSF2 Mut.**	0.16	0.06, 0.48	**0.001**				0.12	0.03, 0.55	**0.006**			
**TP53 Mut.**	2.85	1.55, 5.24	**<0.001**	1.73	0.43, 6.92	0.44	3.45	1.43, 8.35	**0.006**			

[1] HR = Hazard Ratio, CI = Confidence Interval
