# Supplementary material for: DNA Damage Response−Related Proteins Are Prognostic for Outcome in Both Adult and Pediatric Acute Myelogenous Leukemia Patients: Samples from Adults and from Children Enrolled in a Children’s Oncology Group Study
Source: Int J Mol Sci. 2023 Mar 20;24(6):5898. doi: 10.3390/ijms24065898 (PMC10058043; doi:10.3390/ijms24065898)
Supplement: Supplementary file 1 [file ijms-24-05898-s001.zip › Supplemental Tables/Supplemental Table S4.pdf]

**Supplemental Table S4.** Multivariate analysis for CC clusters.

| | Univariate OS (N=340) | | | Multivariate OS (N=340) | | | Univariate CRD (N=223) | | | Multivariate CRD (N=223) | | |
|---|---|---|---|---|---|---|---|---|---|---|---|---|
| **Variable** | **HR**[1] | **95% CI**[1] | **p-value** | **HR**[1] | **95% CI**[1] | **p-value** | **HR**[1] | **95% CI**[1] | **p-value** | **HR**[1] | **95% CI**[1] | **p-value** |
| **Cluster** | | | | | | | | | | | | |
| C1 | 1.00 | — | | 1.00 | — | | 1.00 | — | | 1.00 | — | |
| C2 | 1.33 | 0.94, 1.88 | 0.11 | 0.68 | 0.46, 1.00 | **0.048** | 0.80 | 0.45, 1.42 | 0.44 | 0.40 | 0.20, 0.80 | **0.009** |
| C3 | 1.58 | 1.13, 2.21 | **0.008** | 0.96 | 0.67, 1.37 | 0.82 | 1.44 | 0.87, 2.38 | 0.15 | 1.09 | 0.60, 1.96 | 0.78 |
| C4 | 2.37 | 1.60, 3.53 | **<0.001** | 1.68 | 1.10, 2.57 | **0.016** | 1.69 | 0.88, 3.22 | 0.11 | 0.95 | 0.45, 2.01 | 0.90 |
| **Age (years)** | 1.04 | 1.03, 1.05 | **<0.001** | 1.03 | 1.02, 1.04 | **<0.001** | 1.04 | 1.02, 1.05 | **<0.001** | 1.03 | 1.01, 1.05 | **0.005** |
| **Males** | 1.10 | 0.85, 1.43 | 0.48 | | | | 1.53 | 1.00, 2.34 | **0.048** | 1.38 | 0.88, 2.19 | 0.16 |
| **White (race)** | 1.52 | 1.10, 2.09 | **0.011** | | | | 1.02 | 0.65, 1.60 | 0.93 | | | |
| **Asian (race)** | 0.40 | 0.18, 0.90 | **0.027** | | | | 1.15 | 0.50, 2.63 | 0.74 | | | |
| **Black (race)** | 1.16 | 0.69, 1.95 | 0.58 | | | | 2.08 | 1.08, 4.02 | **0.028** | 2.53 | 1.20, 5.36 | **0.015** |
| **2nd AML** | 3.01 | 2.31, 3.93 | **<0.001** | 2.13 | 1.57, 2.87 | **<0.001** | 2.26 | 1.47, 3.47 | **<0.001** | 1.72 | 1.02, 2.91 | **0.043** |
| **Unfav. Cyto. Risk** | 2.25 | 1.71, 2.95 | **<0.001** | | | | 1.38 | 0.87, 2.19 | 0.17 | | | |
| **Complex Kar.** | 2.40 | 1.80, 3.20 | **<0.001** | 2.12 | 1.55, 2.89 | **<0.001** | 1.95 | 1.20, 3.15 | **0.007** | 2.52 | 1.36, 4.67 | **0.003** |
| **-5/5q-** | 2.94 | 2.01, 4.30 | **<0.001** | | | | 2.55 | 1.23, 5.30 | **0.012** | 0.76 | 0.28, 2.03 | 0.58 |
| **-7/7q-** | 2.37 | 1.68, 3.33 | **<0.001** | 1.44 | 1.00, 2.06 | **0.049** | 1.67 | 0.81, 3.46 | 0.17 | | | |
| **t(8;21)** | 0.48 | 0.25, 0.93 | **0.031** | 0.50 | 0.25, 1.00 | **0.050** | 0.89 | 0.43, 1.83 | 0.75 | | | |
| **Inv 16** | 0.17 | 0.06, 0.47 | **<0.001** | 0.22 | 0.08, 0.60 | **0.003** | 0.31 | 0.11, 0.85 | **0.023** | 0.26 | 0.08, 0.84 | **0.024** |
| **Del 12** | 5.55 | 2.44, 12.7 | **<0.001** | | | | | | | | | |
| **CEBPA Mut.** | 0.62 | 0.39, 0.98 | **0.043** | | | | 0.73 | 0.38, 1.43 | 0.36 | | | |
| **FLT3 Mut.** | 0.54 | 0.37, 0.78 | **0.001** | | | | 0.55 | 0.32, 0.95 | **0.033** | 0.66 | 0.36, 1.23 | 0.19 |
| **MLL Mut.** | 2.01 | 1.17, 3.45 | **0.012** | | | | 2.61 | 1.01, 6.72 | **0.047** | | | |
| **NPM1 Mut.** | 0.56 | 0.38, 0.83 | **0.004** | | | | 0.59 | 0.34, 1.02 | 0.061 | | | |
| **PTPN11 Mut.** | 1.80 | 1.15, 2.79 | **0.009** | | | | 1.76 | 0.87, 3.56 | 0.11 | | | |
| **RUNX1 Mut.** | 1.27 | 0.81, 1.99 | 0.29 | | | | 2.65 | 1.34, 5.26 | **0.005** | | | |
| **SRSF2 Mut.** | 1.57 | 0.55, 4.47 | 0.40 | | | | 49.6 | 4.38, 560 | **0.002** | | | |
| **TP53 Mut.** | 3.55 | 2.49, 5.06 | **<0.001** | | | | 2.34 | 1.21, 4.55 | **0.012** | | | |

[1] HR = Hazard Ratio, CI = Confidence Interval
